# *sox9b* is required in cardiomyocytes for cardiac morphogenesis and function

**DOI:** 10.1038/s41598-018-32125-7

**Published:** 2018-09-17

**Authors:** Joseph C. Gawdzik, Monica S. Yue, Nathan R. Martin, Loes M. H. Elemans, Kevin A. Lanham, Warren Heideman, Ryan Rezendes, Tracie R. Baker, Michael R. Taylor, Jessica S. Plavicki

**Affiliations:** 1 Molecular and Environmental Toxicology Center, University of Wisconsin at Madison, Madison, WI USA; 2 Division of Pharmaceutical Sciences, University of Wisconsin at Madison, Madison, WI USA; 3 Division of Toxicology, Institute for Risk Assessment Sciences (IRAS), Utrecht University, Utrecht, The Netherlands; 4 Wayne State University, Institute of Environmental Health Sciences, Detroit, MI USA; 5 Department of Pathology and Laboratory Medicine, Brown University, Providence, RI USA

## Abstract

The high mobility group transcription factor *SOX9* is expressed in stem cells, progenitor cells, and differentiated cell-types in developing and mature organs. Exposure to a variety of toxicants including dioxin, di(2-ethylhexyl) phthalate, 6:2 chlorinated polyfluorinated ether sulfonate, and chlorpyrifos results in the downregulation of tetrapod *Sox9* and/or zebrafish *sox9b*. Disruption of *Sox9/sox9b* function through environmental exposures or genetic mutations produce a wide range of phenotypes and adversely affect organ development and health. We generated a dominant-negative *sox9b* (*dnsox9b*) to inhibit *sox9b* target gene expression and used the Gal4/UAS system to drive *dnsox9b* specifically in cardiomyocytes. Cardiomyocyte-specific inhibition of *sox9b* function resulted in a decrease in ventricular cardiomyocytes, an increase in atrial cardiomyocytes, hypoplastic endothelial cushions, and impaired epicardial development, ultimately culminating in heart failure. Cardiomyocyte-specific *dnsox9b* expression significantly reduced end diastolic volume, which corresponded with a decrease in stroke volume, ejection fraction, and cardiac output. Further analysis of isolated cardiac tissue by RT-qPCR revealed cardiomyocyte-specific inhibition of *sox9b* function significantly decreased the expression of the critical cardiac development genes *nkx2.5*, *nkx2.7*, and *myl7*, as well as *c-fos*, an immediate early gene necessary for cardiomyocyte progenitor differentiation. Together our studies indicate *sox9b* transcriptional regulation is necessary for cardiomyocyte development and function.

## Introduction

Congenital heart and great vessel defects are the leading cause of infant mortality and morbidity in the United States, yet the etiology of most congenital defects remains unknown^1–4^. Genetic mutations and environmental exposures are often presented as important, but independent, factors that contribute to the development of congenital heart and great vessel defects. This dichotomous presentation persists, in part, from the siloing of scientific disciplines and the corresponding lack of crosstalk between the fields of developmental toxicology and developmental genetics. Significant progress has been made in our understanding of the genetics of cardiovascular development, and many important studies have demonstrated the adverse effects of toxicant exposure on cardiovascular development. However, comparatively few studies have examined how cellular changes induced by environmental exposures intersect with genetic networks known to play critical roles in cardiac development. *SOX9* is a high mobility group (HMG) domain containing transcription factor necessary for establishing and maintaining pools of stem and progenitor cells, and is expressed in multiple cell types in developing and mature organs^5^. Clinical and developmental genetic studies have demonstrated that human *SOX9* and its homologs play critical roles in the development of multiple organ systems including the heart (reviewed in Pritchett *et al*., 2011)^6^. Mutations in human *SOX9* cause Campomelic Dysplasia (CD), a rare haploinsufficiency disorder that is characterized by defective chondrogenesis, 46,XY sex reversal, and usually results in death^7^. CD patients, as well as individuals with mutations in the *SOX9* promoter and 5′UTR, have been reported to have a number of congenital heart and great vessel defects including Tetralogy of Fallot, ventricular and atrial septal defects, patent foramen ovale, and aortic stenosis^8–10^.

In the mouse, *Sox9* function has been most studied in the endocardium, where it regulates the expression of key transcription factors necessary for heart valve formation and is also necessary for maintaining valve health^11,12^. Loss of *Sox9* is embryonic lethal; it is believed that null embryos die as a result of heart failure, though it is not known whether this is due to valve malformations or loss of *Sox9* in other cardiac cell types. Human *SOX9*, mouse *Sox9*, and zebrafish *sox9b* are expressed in embryonic and adult cardiomyocytes, however its function(s) in cardiomyocyte development and function are unknown^13–15^.

Developmental toxicology studies indicate that tetrapod *Sox9* and zebrafish *sox9b* are downregulated following exposure to a number of environmental contaminants including dioxin, di(2-ethylhexyl) phthalate, 6:2 chlorinated polyfluorinated ether sulfonate, and chlorpyrifos^16–20^. These exogenous compounds affect gene expression through hyperactivation of the aryl hydrocarbon receptor (AHR), a ligand activated transcription factor^21^. Ligand-bound AHR translocates from the cytoplasm to the nucleus where it forms a heterodimer with the AHR nuclear translocator (ARNT). Together, the AHR-ARNT complex binds AHR response elements (AREs) in the genome to regulate target gene expression^22,23^. In zebrafish, dioxin exposure results in a suite of heart malformations, a global downregulation of *sox9b* in the developing embryo, and a specific downregulation of *sox9b* in the embryonic heart and jaw and the adult regenerating fin^24–26^. Due to a genome duplication, zebrafish have 3 AHRs (AHR1a, AHR1b, and AHR2) with AHR2 mediating dioxin-induced toxicity^27–30^. It was recently demonstrated that dioxin-induced *sox9b* repression in zebrafish embryos is mediated by the upregulation of a novel long noncoding RNA, *slincR*, that is located adjacent to *sox9b*^31^.

Exposure to the AHR agonist dioxin inhibits the formation of the proepicardial progenitor cells that give rise to the epicardium, the outermost layer of the heart^32^. Failure of the epicardium to form is a critical component of dioxin-induced cardiotoxicity and heart failure. *sox9b* mutants and embryos in which Sox9b function has been impaired using morpholino anti-sense technology do not develop proepicardial progenitor cells^24^. Injections of *sox9b* mRNA into dioxin exposed embryos can rescue the formation of proepicardial progenitor cells^24^. Thus, indicating that *sox9b* has essential functions in cardiac development and is an important molecular mediator of dioxin-induced cardiotoxicity. Given that a diverse set of environmental contaminants disrupt *Sox9* and *sox9b* expression further research is needed to understand how loss of *sox9b* and *Sox9* function contributes to additional endpoints of toxicity during development.

We use the zebrafish model to study how toxicant-induced and genetic disruption of *sox9b* function disrupts development because it provides several distinct advantages. External development of zebrafish embryos allows for passive oxygen diffusion and, consequently, the ability for embryos with severe cardiovascular defects to survive to a larval stage and be studied. Furthermore, zebrafish embryos are transparent, which enables us to assess embryonic cardiac function and visualize cardiovascular development *in vivo*.

To determine the role(s) of *sox9b* in cardiomyocyte development and function and to understand how genetic or toxicant-induced inhibition of *SOX9* function may contribute to the development of additional types of congenital heart defects and impact heart health, we developed a dominant-negative *sox9b* (*dnsox9b*) to inhibit the expression of *sox9b* target genes in a cell-type specific manner. Cardiomyocyte-specific inhibition of *sox9b* function disrupted cardiac morphology, significantly inhibited cardiac function, impaired development of endocardial cushions and prevented formation of the epicardium. Analysis of isolated cardiac tissue demonstrated that cardiomyocyte-specific inhibition of *sox9b* function results in a significant decrease in the expression of critical cardiac development genes, including *nkx2.5*, *nkx2.7*, and *myl7*, as well as *c-fos*, an immediate early gene necessary for cardiomyocyte progenitor differentiation^33,34^. Together, our findings demonstrate that *sox9b* function in cardiomyocytes is essential for myocardial development and subsequent cardiac health. Our study provides genetic tools for elucidating *sox9b* function during development and a foundation to study the contribution of toxicant-induced dysregulation of *SOX9* in congenital defects.

## Results

### Design and Validation of a Dominant-Negative *sox9b* (*dnsox9b*)

**Figure 1:**
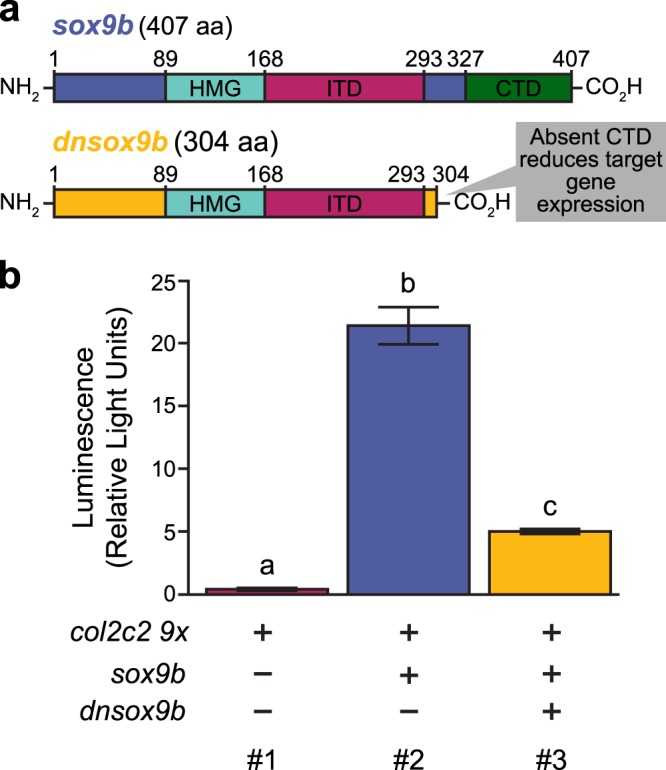
Design and *in vitro* validation of a dominant-negative *sox9b* (*dnsox9b*). **(a)** Schematics of the coding sequences of *sox9b* and *dnsox9b.* The DNA-binding HMG domain (HMG), internal transactivation domain (ITD), and C-terminal transactivation domain (CTD) are indicated. Numbers give amino acid positions. **(b**) *In vitro* HEK293T cell *luciferase* expression assay. Column #1: transfection of the *col2c2-9x* plasmid alone is not sufficient to induce luciferase activity. Column #2: Co-transfection of the *col2c2-9x* and *sox9b-2A-tRFP* plasmids robustly induces *luciferase* activity. Column #3: When *dnsox9b-2A-tRFP* is transfected along with *sox9b-2A-tRFP* and the *col2c2-9x* plasmid, *luciferase* reporter activity is significantly reduced. Average *luciferase* activity is shown relative to a transfection control. Error bars represent standard error of the mean. A one-way ANOVA was used to compare groups. Letters indicate significant differences between groups (p < 0.05), n = 4 for each condition.

Sox9b interacts with DNA through its HMG DNA binding domain and interacts with transcriptional machinery primarily via its C-terminal transactivation (CTD) domain^35^ (Fig. 1a). Truncation of the CTD domain in human SOX9 prevents SOX9 from inducing target gene transcription^36–38^. We generated a truncated *sox9b* that contains the first 304 codons of the *sox9b* coding sequence that retains the HMG DNA binding and internal transactivation domains (ITD), but lacks the CTD (Fig. 1a). Based on the human data, we hypothesized the Sox9b variant would occupy Sox9b binding sites, but would be incapable of facilitating gene transcription. Thus, the variant should behave in a dominant-negative manner and inhibit the expression of *sox9b* target genes.

We linked the dominant-negative *sox9b* variant (*dnsox9b)* to a monomeric tRFP reporter using a “self-cleaving” 2A peptide (*dnsox9b-2A-tRFP*), which allowed the dnSox9b and tRFP proteins to be translated as two separate peptide chains^39^. The construct was fused to the cytomegalovirus (CMV) promoter and cloned into the pDestTol2pA2 expression plasmid for use in a HEK293T cell-based luciferase reporter assay. Concurrently, we generated a plasmid containing full-length *sox9b* (*sox9b-2A-tRFP*) and a plasmid where *luciferase* (*luc*) expression is induced in response to the binding of *sox9b* (*col2c2 9x-luc*). *luc* expression was not induced when HEK293T cells were transfected with the *sox9b* responsive *col2c2 9x-luc* reporter plasmid alone (Fig. 1b, Column #1). In contrast, *luc* expression was robustly induced when the full-length *sox9b-2A-tRFP* plasmid was co-transfected in cells with the *col2c2 9x luc* reporter plasmid (Fig. 1b, Column #2; p < 0.05). When the *dnsox9b-2A-tRFP* plasmid was co-transfected along with the full-length *sox9b-2A-tRFP* plasmid and the *luc* reporter plasmid, the magnitude of *luc* induction was decreased by approximately 64% (Fig. 1b, Column #3; p < 0.05). Thus, indicating that the truncated *sox9b* variant is able to act as a dominant negative as predicted and significantly reduce *sox9b*-mediated transcription.

### Cardiomyocyte-specific *sox9b* Function is Necessary for Cardiac Morphogenesis

**Figure 2:**
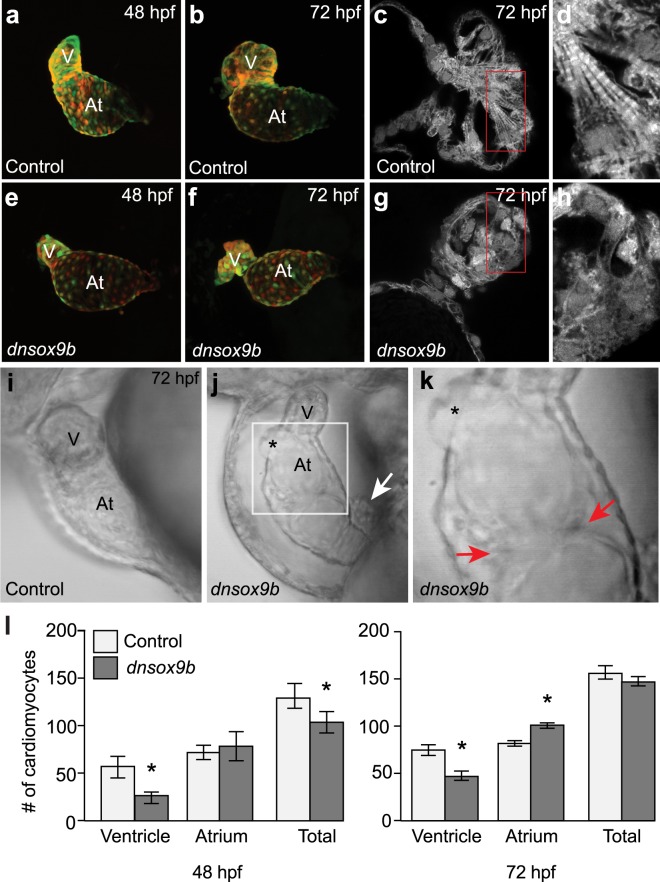
Cardiomyocyte-specific inhibition of *sox9b* function disrupts cardiac morphogenesis. Live control (**a**,**b** and **i**; *Tg(myl7:Gal4VP16;UAS:tRFP)*) zebrafish and zebrafish with cardiomyocyte-specific inhibition of *sox9b* function (**e**,**f**,**j** and **k**; *Tg(myl7:Gal4VP16;UAS:dnsox9b-2A-tRFP)*). **(a,b,e,f)** Embryos and larvae were anesthetized in Tricaine, treated with 20 mM 2,3-butanedione 2-monoxime to temporarily stop heartbeat, then mounted in low melting point agarose for confocal imaging. Ventral images were collected at 48 hpf (**a**,**e**) and 72 hpf (**b**,**f**). Control (*Tg(myl7:Gal4VP16;UAS:tRFP)*) embryos had looped hearts and exhibit time dependent chamber growth. Embryos and larvae with cardiomyocyte-specific inhibition of *sox9b* function (*Tg(myl7:Gal4VP16;UAS:dnsox9b-2A-tRFP)*) had small, compacted ventricles and unlooped heart chambers. **(c,d,g,h)** Super-resolution images of fixed control (**c** and **d**; *Tg(myl7:Gal4VP16;UAS:tRFP)*) and experimental larvae (**g**,**h**; *Tg(myl7:Gal4VP16;UAS:dnsox9b-2A-tRFP)*). Cardiomyocyte-specific inhibition of *sox9b* function disrupted the development of myofibrillar bundles and z-lines were notably absent. **(i–k)** Still images from Supplemental Movies. **(k)** Boxed area in j. Pericardial edema, holes in the heart (asterisks in j,k), and ectopic endothelial cushions (red arrows in k) were observed in larvae with cardiomyocyte-specific inhibition of *sox9b* function. Proepicardial cells were observed adjacent to the myocardium (white arrow in j). **(l)** Quantification of ventricular and atrial cardiomyocytes at 48 and 72 hpf. Cardiomyocyte-specific inhibition of *sox9b* function resulted in a decrease in ventricular cardiomyocytes and an increase in atrial cardiomyocytes. Light bars indicate control group and dark bars indicate the experimental group. Asterisks indicate significant differences between groups as determined by Student’s t-test (p < 0.05), n = 8–10 per group. Ventricle (V) and atrium (At) are abbreviated as indicated.

To determine how inhibiting *sox9b* target gene expression in cardiomyocytes affects zebrafish cardiac development, we employed the Gal4/UAS system to drive uniform *dnsox9b* expression specifically in cardiomyocytes^40^. We generated the following stable, independent transgenic lines: *Tg*(*UAS*:*dnSox9b-2A-tRFP; myl7:EGFP*) and, as a control, *Tg*(*UAS:tRFP; myl7:EGFP)*. Both of the UAS lines carry a cardiomyocyte-specific transgenesis marker (*myl7:EGFP*), which also serves as an additional means of visualizing the developing heart. A cardiomyocyte specific Gal4 line, *Tg(myl7:Gal4VP16)*, was crossed to either the control or experimental UAS line. The corresponding tRFP signal was detected in the developing embryonic heart by approximately 30 (hours post fertilization) hpf and overlaps exclusively with *myl7-*driven EGFP transgene expression in both the atrium and ventricle (Fig. 2). Embryos and larvae with cardiomyocyte-specific *dnsox9b* expression (*Tg(myl7:Gal4VP16;UAS:dnsox9b-2A-tRFP)*) were examined at 48 and 72 hpf for cardiac malformations and compared with control embryos and larvae (*Tg(myl7:Gal4VP16;UAS:tRFP)*). At 48 hpf, the control embryos appear phenotypically normal with well-defined, looped heart chambers (Fig. 2a). In contrast, the embryos with cardiomyocyte-specific *dnsox9b* expression had a small, compacted ventricle, a distended, enlarged atrium, pericardial edema, and an impairment in chamber looping (Fig. 2e). At 72 hpf, the control larvae again appear phenotypically normal with the cardiac chambers continuing to grow and remaining looped (Fig. 2b,i). In contrast, the cardiac malformations observed in larvae with cardiomyocyte-specific *dnsox9b* expression had increased in severity. The ventricle appears proportionally even smaller and the atrium further enlarged. The chambers were unlooped and we observed considerable pericardial edema (Fig. 2f,j).

We used super-resolution confocal microscopy to visualize cardiomyocyte ultrastructure and found that inhibition of *sox9b* function disrupted the development of myofibrillar bundles. Z-lines were notably absent in the cardiomyocytes expressing the dominant negative construct (compare Fig. 2 panels c and d with g and h). In addition, in a subset of larvae, we observed holes in the heart (Fig. 2j,k; Supplemental Movie 4) and what appeared to be endocardial cushions forming in the atrium (red arrows in Fig. 2j,k).

We quantified the number of ventricular and atrial cardiomyocytes in hearts from control animals and animals with cardiomyocyte-specific *dnsox9b* expression at 48 and 72 hpf. Expression of *dnsox9b* in cardiomyocytes resulted in a significant decrease in ventricular cardiomyocytes at 48 and 72 hpf (Fig. 2l). A significant increase in atrial cardiomyocytes was observed at 72 hpf. Together, these findings indicate that *sox9b* function in cardiomyocytes is necessary for cardiomyocyte development, chamber specific growth, and cardiac morphogenesis.

### Cardiomyocyte Expression of *dnsox9b* Impairs Cardiac Function

**Figure 3:**
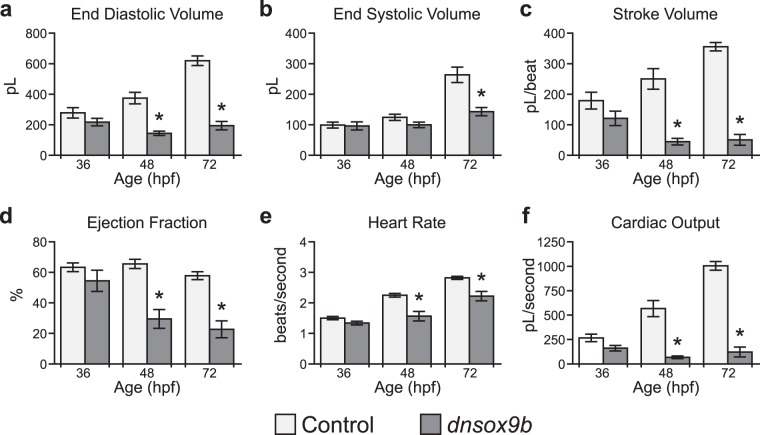
*sox9b* function is necessary in cardiomyocytes for proper cardiac function. **(a–f**) Videos of control (*Tg(myl7:Gal4VP16;UAS:tRFP)*) zebrafish and zebrafish with cardiomyocyte-specific loss of *sox9b* function (*Tg(myl7:Gal4VP16;UAS:dnsox9b-2A-tRFP*) were collected and cardiac function was analyzed at 36, 48, and 72 hpf. End-diastolic (EDV) and end-systolic (ESV) volumes were approximated by applying “The Method of Discs” or “Simpson’s Method” to still images of ventricles at peak diastole and systole. EDV and ESV were used to calculate stroke volume (SV; SV = EDV − ESV) and ejection fraction (EF; EF = (EDV − ESV)/EDV × 100). Heart rate was also measured and, along with stroke volume, used to calculate cardiac output (CO; CO = SV x HR). Error bars represent standard error of the mean. Asterisks indicate significant differences between groups as determined by Student’s t-test. (p < 0.05), n = 7 per group. Light bars indicate control group and dark bars indicate the experimental group.

To determine the effects of cardiomyocyte*-*specific *dnsox9b* expression on cardiac function, we collected movies of beating embryonic and larval hearts and measured ventricular volumes at peak diastole and systole. Ventricular end diastolic volume (EDV) and end systolic volumes (ESV) were used to calculate stroke volume (SV) and ejection fraction (EF). Heart rate was also measured and, along with stroke volume, used to calculate cardiac output (CO)^41–43^. Control (*Tg(myl7:Gal4VP16;UAS:tRFP)*) embryos exhibited time-dependent increases in all cardiac measurements, which corresponded with cardiac growth and maturation between 36–72 hpf (Fig. 3a–f). By 48 hpf, embryos with cardiomyocyte-specific *dnsox9b* expression (*Tg(myl7:Gal4VP16;UAS:dnsox9b-2A-tRFP*) had significantly decreased EDV such that the volume of blood that filled the ventricle at its most relaxed state (peak diastole) was less than half of the volume of blood present in the ventricle of a control embryo (Fig. 3a). This significant decrease in end diastolic volume contributed to a significant decrease in stroke volume and ejection fraction in embryos with cardiomyocyte-specific inhibition of *sox9b* function (*Tg(myl7:Gal4VP16;UAS:dnsox9b-2A-tRFP)*) (Fig. 3c,d). Heart rate was also decreased and, when coupled with the decreased end diastolic volume, resulted in reduced cardiac output (Fig. 3e,f). By 72 hpf, all measured cardiac function parameters were significantly decreased in larvae with cardiomyocyte-specific inhibition of *sox9b* function (*Tg(myl7:Gal4VP16;UAS:dnsox9b-2A-tRFP)*) relative to controls (*Tg(myl7:Gal4VP16;UAS:tRFP)*) (Fig. 3a–f). In the control samples, the heart beats regularly and blood flows in a unidirectional manner from the common cardinal vein into the atrium to the ventricle and out the bulbous arteriosus/outflow tract. The ventricle contracts and relaxes in the control samples, which is reflected in the change in ventricular size (see Supplemental Movies 1 and 2). In larvae expressing cardiomyocyte-specific *dnsox9b*, contraction occurred as a peristaltic-like wave that propagated from the atrium to the ventricle and there was little change in ventricular size (see Supplemental Movies 3–6). Additionally, the outflow tract was compacted to the extent that at 72 hpf blood flow through the heart and into systemic circulation was blocked (compare Supplemental Movies 1 and 2 with Supplemental Movies 3–5). Cardiomyocyte-specific inhibition of *sox9b* function ultimately results in cardiac failure and death.

### Inhibition of *sox9b* Function in Cardiomyocytes Impairs Endocardial Development

**Figure 4:**
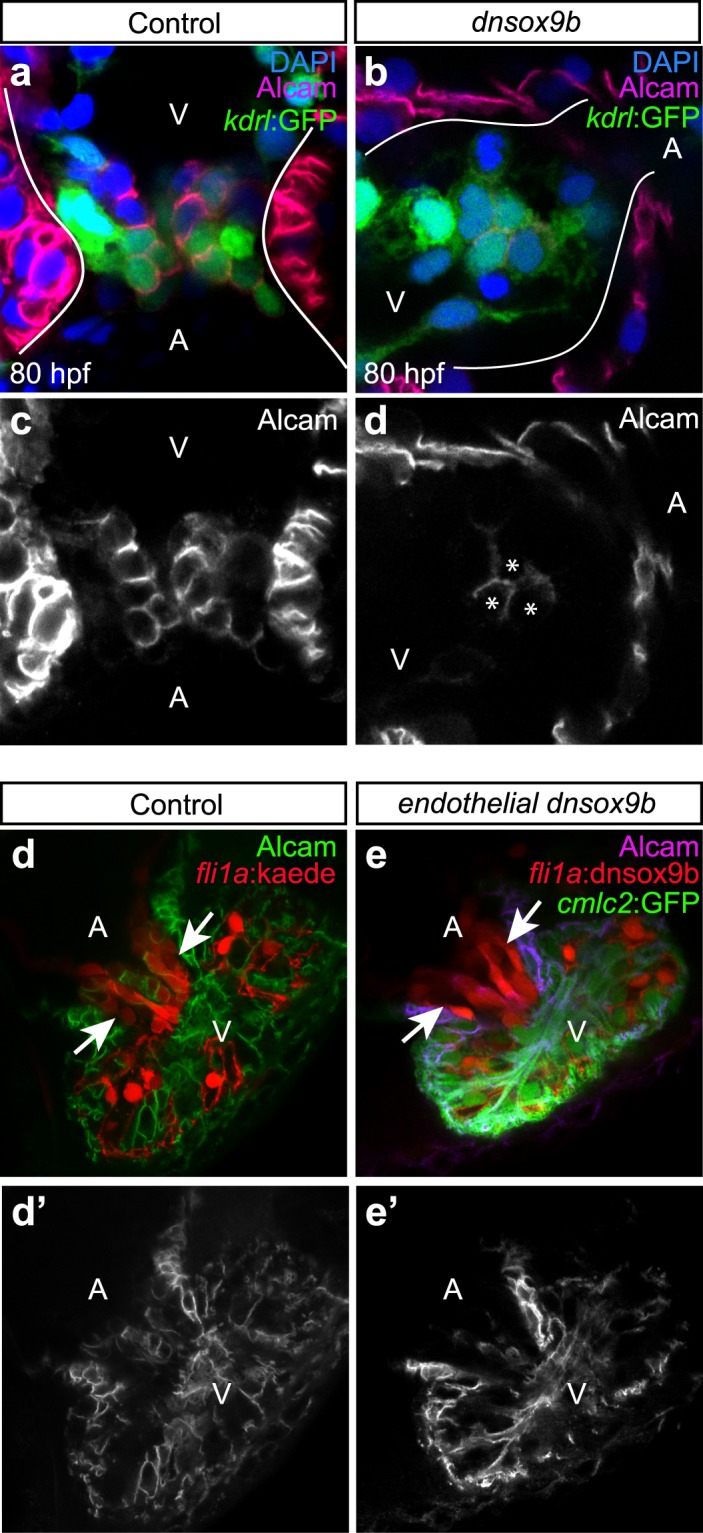
Cardiomyocyte-specific but not endothelial-specific inhibition of sox9b function results in hypoplastic atrioventricular cushions. Control larvae (**a**,**c**,**d**,**d**’; *Tg(myl7:Gal4VP16;kdrl:GFP)*) and larvae with cardiomyocyte-specific loss of *sox9b* function (**b**,**d**,**e**,**e’**; *Tg(myl7:Gal4VP16;kdrl:GFP;UAS:dnsox9b-2A-tRFP)*) were fixed at 80 hpf and processed for fluorescent immunohistochemistry using antibodies against activated leukocyte cell adhesion molecule (Alcam; purple). **(a,b)** Larvae were mounted ventrally in low melting point agarose and imaged at 40x magnification with a confocal microscope. **(c,d)** Boxed areas in a & b showing endocardial expressing Alcam, a marker of differentiated endocardial cushion cells. (**a**,**c**) AV cushions form normally in control larvae, as indicated by a coalescence of Alcam + endocardial cells at the junction between the atrium and ventricle (boxed area in a). (**b**,**d**) Larvae with in cardiomyocyte-specific inhibition of *sox9b* had hypoplastic cushions with Alcam + endocardial cells. White arrow indicates endocardium pushing through a hole in myocardium. Ventricle (V) and atrium (At) are abbreviated as indicated. Images are representative phenotypes, n = 5 per group. **(e-f’**) Control larvae (*Tg(fli1a:Gal4ff;UAS:Kaede)*) and larvae with endothelial-specific loss of *sox9b* function *Tg(fli1a:Gal4ff;UAS:dnsox9b-2A-tRFP; UAS:Kaede)*) were fixed at 120 hpf, processed for fluorescent immunohistochemistry using antibodies against Alcam, and scored for the presence of endothelial cushion. Endothelial cushions clearly formed in both control larvae (e) and larvae with endothelial-specific loss of *sox9b* function (f). Images are representative phenotypes, n = 8 per group.

Global loss of *sox9b* function disrupts endocardial cushion formation and therefore cardiac valve morphogenesis^24^. To investigate whether cardiomyocyte-specific *dnsox9b* expression impairs atrioventricular (AV) cushion formation, we generated a cardiomyocyte-specific Gal4 line with an endothelial reporter (*Tg(myl7:Gal4VP16;kdrl:GFP)*) and crossed this line to a *Tg(UAS:dnsox9b-2A-tRFP)* line that carries a *crystallin alpha:mCherry* (*cryA:mCherry*) transgenesis marker. We used both live images and fixed samples to analyze the morphology of the developing AV cushion at 80 hpf. With fixed samples, we performed fluorescent immunohistochemistry with an activated leukocyte cell adhesion molecule (Alcam) antibody, a marker of differentiated endocardial cushion and myocardial cells, to assess endocardial cushion development. Differentiated endocardial cushion cells were identified by the presence of *kdrl* driven GFP, the presence of Alcam staining, and cuboidal morphology. In control (*Tg(myl7:Gal4VP16;kdrl:GFP*) embryos and larvae, differentiated endocardial cells had coalesced to form both inferior and superior AV cushions (Fig. 4a,c). Differentiated Alcam + endocardial cushion cells were also present at the AV junction in embryos and larvae with cardiomyocyte-specific *dnsox9b* expression. However, there were fewer endocardial cushion cells and, consequently, the cushions were hypoplastic relative to controls (Fig. 4c,d). Together, these findings indicate that changes in myocardial *sox9b* function alter the development of the endocardium either directly from cell-cell interactions or indirectly through changes in cardiac function.

### Inhibition of *sox9b* Function in the Endocardium Does Not Impair Endocardial Cushion Development

Next, we examined whether loss of *sox9b* function specifically in endothelial cells was sufficient to inhibit endocardial cushion formation. To do so, we crossed our *Tg(UAS:dnsox9b-2A-tRFP)* line to an endothelial Gal4 line (*Tg(fli1a:Gal4FF*^*ubs3*^*; UAS:Kaede)*) and scored fixed, Alcam stained samples at 120 hpf. Endocardial cushions clearly formed in both control larvae and larvae with endothelial-specific loss of *sox9b* (Fig. 4).

### Cardiomyocyte-Specific *dnsox9b* Expression Impairs Epicardium Formation

**Figure 5:**
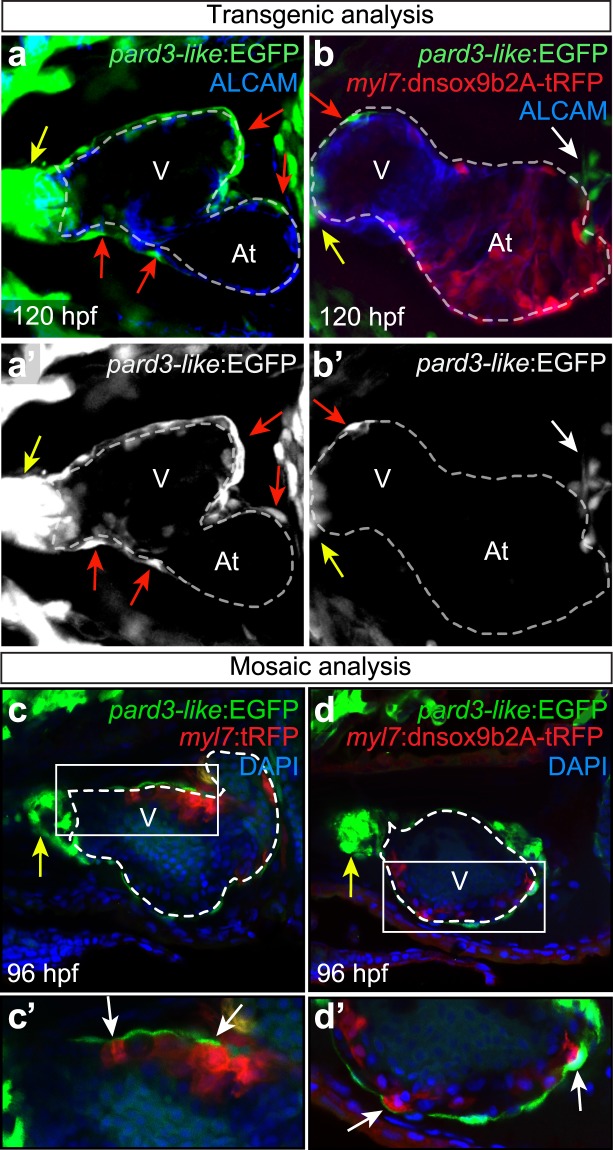
Impaired epicardium formation following cardiomyocyte-specific inhibition of *sox9b* function. Control (*Tg(myl7:Gal4VP16;pard3-like:EGFP)*) larvae and larvae with cardiomyocyte-specific loss of *sox9b* function (*Tg(myl7:Gal4VP16;pard3-like:EGFP;UAS:dnsox9b-2A-tRFP)*) were fixed at 120 hpf and processed for fluorescent immunohistochemistry using antibodies against tRFP (red) and Alcam (blue). Larvae were mounted ventrally in low melting point agarose and imaged at 40x magnification with a confocal microscope. The myocardium is indicated by a dashed line. (**a**,**a’**) The epicardium forms normally in control larvae, as indicated by EGFP-labeled epicardial cells on the surface of the ventricular and atrial myocardium (red arrows). (**b**,**b’**) Epicardium formation is impaired when *sox9b* function is lost in cardiomyocytes. Very few EGFP-labeled epicardial cells can be seen on the ventricular myocardium (red arrow) and none are present on the atrial myocardium. A cluster of proepicardial progenitors is visible in the pericardial space (white arrow). (**c**,**d**) *pard3-like:EGFP* zebrafish embryos were injected with either a control plasmid (*Tg(myl7:tRFP*)) or plasmid with the *dnsox9b* fused to a cardiomyocyte specific promoter (*Tg(myl7:dnsox9b-2A-tRFP*)). Injections resulted in mosaic expression of the constructs in cardiomyocytes. Samples were fixed at 96 hpf and processed for immunohistochemistry using an antibody for tRFP (red) and DAPI to label nuclei (blue). With mosaic *dnsox9b* expression, epicardial cells (red arrows) are found overlying dnSox9b^+^ myocardial cells. Ventricle (V) and atrium (At) are abbreviated as indicated, and the outflow tract (bulbus arteriousus) is indicated by the yellow arrow. Images are representative phenotypes, n = 8 per group.

Global loss of *sox9b* function inhibits proepicardial progenitor cell specification and, consequently, the outmost layer of the heart, the epicardium, does not form in *sox9b* mutants and morphants^24^. To determine whether epicardial development was altered by cardiomyocyte-specific *dnsox9b* expression, we crossed the cardiomyocyte-specific Gal4 (*Tg(myl7:Gal4VP16*) line to an enhancer trap line that, among other tissues, marks the epicardium and outflow tract (ET27 or *pard3-like:EGFP*) in the developing heart. The cardiomyocyte-specific Gal4 line with the epicardial reporter (*Tg(myl7:Gal4VP16;pard3-like:EGFP*) was then crossed to the *Tg(UAS:dnsox9b-2A-tRFP)* and *Tg(UAS:tRFP)* lines carrying a *cryA:mCherry* transgenesis marker to determine if cardiomyocyte-specific *dnsox9b* expression altered proepicardium or epicardium development. Using brightfield microscopy, we determined that expression of *dnsox9b* in the myocardium did not affect the proepicardial progenitor cell specification or clustering (Fig. 2j and Supplemental Fig. 1). To assess whether the epicardium subsequently formed, control (*Tg(myl7*:*Gal4VP16;pard3-like:EGFP)*) and experimental (*myl7:Gal4VP16;pard3-like:EGFP*; *UAS*:*dnsox9b-2A-tRFP)* larvae were collected at 120 hpf, stained with antibodies against Alcam to visualize gross cardiac morphology, and then imaged using confocal microscopy. In control larvae, EGFP-labeled epicardial cells can be seen overlying the Alcam^+^ (blue) myocardial cells on both the ventricle and atrium, forming a clear epicardial layer (Fig. 5a,a’). In contrast, few epicardial cells were present on the ventricle of larvae expressing the cardiomyocyte-specific *dnsox9b-2A-tRFP* (Fig. 5b,b’). Since myocardial inhibition of *sox9b* function did not alter proepicardial progenitor cell development, the failure of the epicardium to form is likely due to the failure of proepicardial progenitor cells to migrate to the myocardium. Cardiac function is necessary for proepicardial cell migration and cardiac function is significantly impaired in larvae with cardiomyocyte-specific *dnsox9b* expression^44^. Therefore, the observed loss of the epicardium in larvae with cardiomyocyte-specific loss of *sox9b* function may be secondary to changes in cardiac function.

To address whether disruptions in cardiac function contribute to the failure of the epicardium to form in larvae with cardiomyocyte-specific *dnsox9b* expression, we generated larvae that had a subset of cardiomyocytes expressing the *dnsox9b* construct, but normal heart function. To do so, we fused the *dnsox9b-2A-tRFP* construct to a cardiomyocyte-specific (*myl7*) promoter. The resulting *myl7:dnsox9b-2A-tRFP* and the corresponding control (*myl7:tRFP*) constructs were cloned into the pDestTol2pA2 expression vector and injected into zebrafish embryos at the one-cell stage. The injections produced mosaic expression of the constructs in larval hearts with normal heart function. In both the control and experimental groups, proepicardial cells successfully migrated to the myocardium as evidenced by the presence of epicardial cells at multiple sites on the myocardium (Fig. 5c,d). Next, we examined whether the epicardial cells could be observed overlying cardiomyocytes expressing the *dnsox9b* construct and found that expression of the *dnsox9b* or control construct did not impact epicardium formation (Fig. 5c’,d’). Thus, providing further support for the interpretation that disruptions in cardiac function impair proepicardial cell migration and drive the epicardial phenotypes observed during analysis of the non-mosaic transgenic lines.

### Myocardial Inhibition of *sox9*b Function Reduces the Expression of Critical Cardiac Development Genes

**Figure 6:**
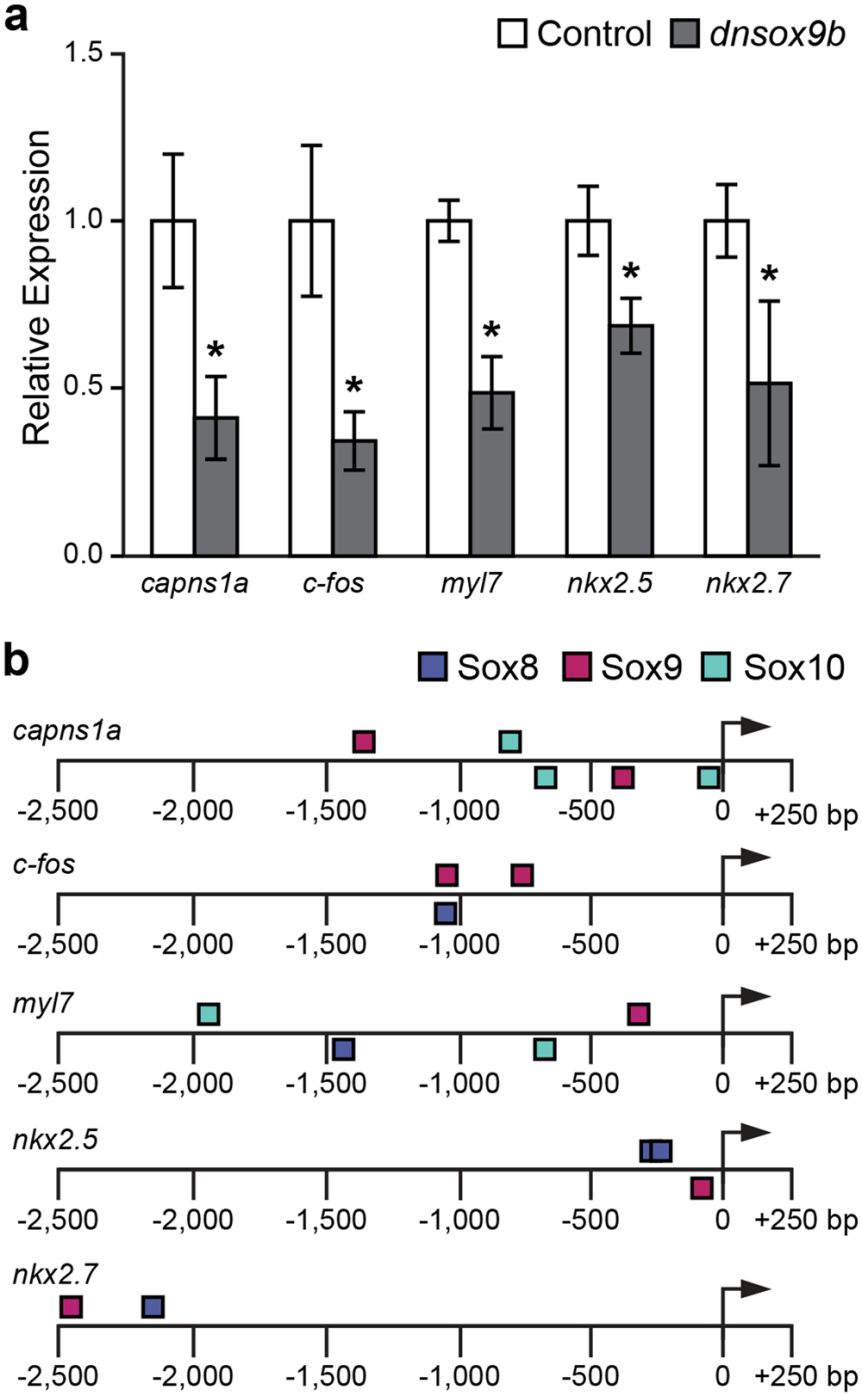
Cardiomyocyte-specific inhibition of *sox9b* function alters the expression of critical cardiac development genes. **(a)** Expression of selected genes from hearts isolated from control (*Tg(myl7:Gal4VP16;UAS:tRFP)*) embryos and embryos with cardiomyocyte-specific loss of sox9b function (*Tg(myl7:Gal4VP16;UAS:dnsox9b-2A-tRFP))* at 50 hpf. Expression of each transcript was analyzed using RT-qPCR normalized to *actb1*. The expression of these genes in control samples was set to 1. Asterisks represent a significant difference in expression between control (light gray) and experimental hearts (dark gray), *p < 0.05 and False Discovery Rate (FDR) < 0.25, n = 4 for each group. Error bars represent standard error of the mean. **(b)** A schematic depicting the −2.5 kb upstream DNA sequence of genes in panel A shows the locations of SOX8, SOX9, and SOX10 binding sites in this region. The sequence is shown as a solid black line with distance relative to the TSS (arrow) indicated. The position of each oval above or below the sequence refers to the strand each binding site resides on (top = positive strand, bottom = minus strand).

Next, we used RT-qPCR to validate our *dnsox9b* construct *in vivo* and to examine potential genetic mechanisms mediating the observed phenotypes. We isolated whole hearts at 50 hpf from control (*Tg(myl7:Gal4VP16;UAS:tRFP)*) embryos and embryos with cardiomyocyte-specific inhibition of *sox9b* function (*Tg(myl7:Gal4VP16;UAS*:*dnsox9b-2A-tRFP)*). We examined the expression of a panel of genes including several genes previously identified to be regulated by Sox9 in other cell-types, as well as genes known to be important for cardiac development (Supplemental Fig. 2, Supplemental Table 2). Two genes known to be regulated by Sox9 in mouse embryonic limb buds and chondrocytes, *col1a2*^12^ and *col2a1*^45^, respectively, were expressed at very low levels in the developing heart and were found to not be significantly affected by *dnsox9b* expression (Supplemental Fig. 2, Supplemental Table 3). We found expression of *dnsox9b* in the zebrafish myocardium resulted in a significant downregulation of *c-fos* and *capns1a*, genes known to be Sox9 targets in mouse chondrocytes^46^ (Fig. 6a).

We initially intended to assess the expression of the *dnsox9b* construct relative to endogenous *sox9b* expression in cardiomyocytes. However, the gene expression probe used to measure *sox9b* mRNA levels targets a region of the *sox9b* coding sequence that was retained in the *dnsox9b-2A-tRFP* construct (5’ of codon 304) and, as a result, the probe detected both endogenous *sox9b* and the *dnsox9b* construct. Therefore, we detected significantly higher levels of this retained portion of the *sox9b* coding sequence in embryos overexpressing the *dnsox9b-2A-tRFP* construct compared to control embryos (Supplemental Fig. 2).

Given that the reduction in ventricular size and the corresponding increase in atrial size observed following myocardial inhibition of *sox9b* function is reminiscent of phenotypes observed in *nkx2.5* mutants and *nkx2.5; nkx2.7* double mutants, we asked whether impairing Sox9b function in cardiomyocytes affected *nkx2.5* and *nkx2.7* expression. *nkx2.5* is expressed early in the primary and secondary heart fields and expression needs to be maintained in order for ventricular cardiomyocytes to maintain their identity. We found that cardiomyocyte-specific inhibition of *sox9b* function significantly decreased the expression of *nkx2.5*, *nkx2.7* and *myl7*, indicating that impairing *sox9b* function can disrupt the expression of critical cardiac development genes (Fig. 6a).

To address whether the downregulated genes may be direct targets of Sox9b, we used MatInspector^47^ to search for Sox9 binding sites in the promoter regions of *capns1a*, *c-fos*, *myl7*, *nkx2.5*, and *nkx2.7* (Fig. 6b). We examined the DNA sequence −2.5 kb upstream of the transcriptional start sites (TSS) and found Sox9 binding sites were present in the promoter regions for all five genes (Fig. 6b). Furthermore, the promoter regions also contained binding sites for Sox8 and Sox10, which are members of the SoxE family of transcription factors and form heterodimers with Sox9^48^ (Fig. 6b). Additional studies are necessary to determine whether *capns1a*, *c-fos*, *myl7*, *nkx2.5*, and *nkx2.7* are directly or indirectly regulated by Sox9b.

## Discussion

Several recent studies have demonstrated a link between developmental exposure to different types of environmental contaminants and a reduction in mouse *Sox9* and/or zebrafish *sox9b* expression^16–18^. *SOX9* and its homologs are both targets and mediators of canonical WNT, TGFβ, and fibroblast growth factor (FGF) signaling pathways, which are essential for organogenesis^49^. We postulate that the intersection of *Sox9* and *sox9b* with critical signaling pathways makes it a common target of multiple toxicants during development. Here we describe transgenic tools for spatially controlling *sox9b* function during development. The developed transgenic lines provide a means for identifying the cell-types mediating toxicant induced phenotypes and for studying the mechanisms by which reduced *sox9b* expression causes developmental malformations. We used the described transgenic lines to manipulate *sox9b* function specifically in cardiomyocytes and identified new critical functions for *sox9b* in cardiomyocyte development and function as well as potential downstream targets mediating a subset of the observed phenotypes.

Using both *in vitro* and *in vivo* approaches, we demonstrate that the generated *dnsox9b* variant attenuates *sox9b* transcriptional regulation. The observed reduction of target gene expression attained with the zebrafish *dnsox9b* variant is consistent with previous reports of target gene inhibition following overexpression of a human SOX9 variant lacking a CTD^36,37^. Additional factors may also contribute to the observed changes in gene expression. The C-terminal portion of Sox9 has been shown to be sumoylated, which can also affect Sox9 regulation of target gene expression^50^. Since the dnSox9b lacks a C-terminal domain, we do not know how potentially interfering with post-translational modification affects dnSox9b binding and protein interactions. Nevertheless, the decrease in Sox9b target gene expression by the *dnsox9b* variant both *in vitro* and *in vivo* validate the *dnsox9b* construct as an effective tool for blocking Sox9b target gene expression.

Previous research demonstrated that zebrafish exposed to dioxin during early embryogenesis have severe cardiac malformations, decreased cardiac function, and a reduction in cardiac *sox9b* expression^24,41,51^. Knock-down of *sox9b* mRNA using morpholino oligonucleotides recapitulates many of the dioxin-induced cardiotoxic phenotypes and injections of *sox9b* mRNA rescue the formation of proepicardial progenitor cells. Thus, suggesting some causality. However, both dioxin exposure and morpholinos reduce *sox9b* expression throughout the entire zebrafish embryo as well as in heart tissue. Therefore, it was not known if the observed phenotypes were the result of *sox9b* expression being lost from one primary cardiac cell-type or a combination of cell-types in the heart or even non-cardiac tissues. We generated transgenic tools to determine if inhibiting *sox9b* function specifically in cardiomyocytes could recapitulate the cardiac phenotypes seen in zebrafish embryos following dioxin exposure. We found that, indeed, cardiomyocyte-specific inhibition of *sox9b* function recapitulated many of the phenotypes observed following dioxin exposure as well as those seen with global loss of *sox9b* function including: pericardial edema, chamber unlooping, impaired cardiac function, hypoplastic endocardial cushions, and heart failure.

Next, we examined whether endothelial-specific loss of *sox9b* was sufficient to disrupt endocardial cushion formation. We found that endocardial cushions formed and functioned normally in larvae with endothelial-specific loss of *sox9b* function. However, in the experiments we performed, the endothelial-specific Gal4 was driving both a UAS dnSox9b and a UAS reporter construct (*Tg(UAS:Kaede*). It is possible that the Gal4 was diluted and the dnSox9b was less effective. Additional studies are necessary to determine whether s*ox9b* has roles in later stages of zebrafish valve development such as valve leaflet development or maintaining valve health.

Cardiomyocyte-specific inhibition of *sox9b* function inhibited epicardium formation: while the proepicardial progenitor cells that give rise to the epicardium did form, these epicardial cells did not migrate onto the myocardium. Since proepicardial progenitor cell migration is dependent on cardiac function the observed inhibition of epicardium formation is likely secondary to changes in cardiac function^44^. Global inhibition of *sox9b* with the *dnsox9b* construct did inhibit proepicardial progenitor cell formation (data not shown), which is consistent with previous reports of requirement for *sox9b* in proepicardial progenitor cell formation^24^. Between 48 and 72 hpf, the cardiac malformations in embryos and larvae with cardiomyocyte-specific inhibition of *sox9b* function become progressively more severe. Hemodynamic forces and shear stress produced by blood moving through the heart are necessary for proper cardiac morphogenesis. Impaired cardiac function can produce morphological changes that cause further dysfunction. This cycle likely repeats until complete heart failure results.

Since *sox9b* transcriptional targets in the developing zebrafish heart had not previously been identified, we created an *a priori* list of candidate genes based on previous studies identifying *Sox9* targets in other cell-types^12,46,52^. The list also included known cardiac development genes with loss of function phenotypes that resembled the *dnsox9b* loss of function phenotypes (Supplemental Table 2). We observed a significant reduction in *c-fos*, *capns1a, nkx2.5*, *nkx2.7*, and *myl7* specifically in cardiac tissue following loss of *dnsox9b* function. In addition, we identified Sox9 binding sites in upstream regions of these genes as well as binding sites for Sox8 and Sox10, which are known to form heterodimers with Sox9^48^ (Fig. 6). Sox9 has been shown to directly bind to the *c-fos* promoter in the mouse atrioventricular canal and limb bud as well as in hair follicle stem cells^12,46,52,53^. In addition, *c-fos* has been implicated in cardiomyocyte progenitor differentiation and is necessary for cardiomyocyte proliferation following acute injury in the embryonic and adult zebrafish heart, respectively^33,34^. Sox9 binds to the *Capns1* promoter in neonatal mouse chondrocytes^46,53^. The *capns1a* gene encodes a member of the Calpain family of cysteine proteases, which regulate integrin-mediated cell migration^54,55^. These proteases may play an important role in heart development as mice with a disrupted variant of *Capn4* die mid-gestation and show cardiac defects as well as hemorrhaging^56^. Taken together, reductions in either *c-fos* or *capns1a* expression could significantly contribute to the altered morphology and impaired cardiac function observed following cardiomyocyte-specific *dnsox9b* expression.

Expression of *nkx2.5*, *nkx2.7*, and *myl7* (*cmlc2*) were also reduced as a result of cardiomyocyte-specific *dnsox9b* expression. The homeobox genes *nkx2.5* and *nkx2.7* are essential for cardiac morphogenesis and survival. In humans, mutations in *NKX2.5* have been associated with numerous congenital heart defects including Tetralogy of Fallot and Ebstein’s anomaly^57,58^. *myl7* is another critical cardiac development gene and is necessary for cardiac sarcomere assembly and cardiac function^59,60^. Disruptions in these critical cardiac development genes could play a substantial role in the cardiac malformations observed following inhibition of *sox9b* function. The observed changes in gene expression were detected at 50 hpf, a time at which *nkx2.5* and *nkx2.7* expression is confined to the ventricle. Given that there are fewer ventricular cardiomyocytes following cardiomyocyte-specific loss of *sox9b* function, the changes in *nkx2.5* and *nkx2.7* could be due to the observed changes in ventricular development. However, we did detect Sox9 and SOXE binding sites in the promoters of *nkx2.5, nkx2.7, and myl7*, which suggests these genes could be directly regulated by *sox9b*. Additional studies are necessary to determine whether the downregulated genes are indeed *sox9b* targets.

In humans, mutations upstream and within *SOX9* have been linked to congenital heart and vessel defects including Tetralogy of Fallot, ventricular and atrial septal defects, patent foramen ovale, and aortic stenosis^8,61^. The reported clinical phenotypes indicate *SOX9* plays a number of roles in human cardiac development. Furthermore, since loss of *SOX9* frequently results in embryonic lethality the observed clinical phenotypes likely reflect the less severe phenotypes associated with human loss of *SOX9* function. We anticipate that mutations that affect cardiomyocyte development and function would result in embryonic or perinatal death. The displaced atrial cushions seen in our studies are reminiscent of Ebstein’s Anomaly, a rare congenital defect seen in humans for which the developmental origins are unknown. Cases of Tetralogy of Fallot and Ebstein’s Anomaly in human infants have also been associated with maternal exposure to organic solvents, insecticides, herbicides and fungicides^62,63^. Together, these findings support a link between environmental exposures, reduced *sox9b* function, and heart malformations.

The developed transgenic tools presented here can be used to gain further insights into the mechanisms by which environmental contaminants that reduce *sox9b* function subsequently cause developmental malformations. The *sox9b* expression pattern encompasses numerous cell-types during development and continues to be expressed in adult organs and structures. Using the GAL4/UAS system, the *Tg(UAS:dnsox9b)* line can be used to impair *sox9b* function in different tissues and, when paired with inducible Gal4 lines, researchers can also gain temporal control to assess loss of function phenotypes in adult animals. These approaches can be used to study whether cell-type-specific inhibition of *sox9b* function is sufficient to recapitulate specific developmental malformations following toxicant exposures. The developed tools can be broadly applied by developmental toxicologists and developmental biologists to understand *sox9b* function and toxicant-induced dysregulation of *sox9b* on the development of other organ systems and adult organ health.

## Materials and Methods

### Oligonucleotides

All primers were synthesized from Integrated DNA Technologies (Coralville, IA). Primer sequences are available in Supplemental Table 1.

### Plasmid Construction

The *pGL2 col2c2-9x* Luciferase Reporter Vector was constructed from a synthetic DNA segment containing the *col2c2* enhancer^45^ flanked by 5′ BglII and 3′ BamHI restriction sites. Following digestion with BglII and EcoRI, the *col2c2* enhancer was ligated into the *pGL2-Basic* Luciferase Reporter Vector (Promega), this process was repeated three times to generate *pGL2 col2c2-3x*. The *col2c2-3x* enhancer was amplified using the pGL2 *col2c2-3x* fwd and rev primers and the *pGL2 col2c2-3x* as a template. This amplicon was gel purified, digested with BglII and BamHI, and then ligated into *pGL2 col2c2-3x* twice, to obtain the *pGL2 col2c2-9x* Reporter Vector. Insertion of the *col2c2-9x* sequences was confirmed by DNA sequencing. The *E1b* TATA box and carp beta-actin 5′-UTR fragment^64,65^ was amplified from the p5E-UAS entry vector^66^, using *E1b minimal promoter HindIII* fwd and rev primers, digested with HindIII, then ligated into the *pGL2 col2c2-9x* vector. All PCR reactions were performed using *Taq* DNA Polymerase (Invitrogen).

The *pME:sox9b* no stop and *pME*:*dnsox9b* entry vectors were made by amplifying the *sox9b* coding sequence (NM_131644) from the *pCMV-Sport6 sox9b* plasmid (Open Biosystems, Huntsville, AL) primed with *attB1 sox9b ORF* fwd and *attB2r sox9b no stop* rev or *attB2 sox9b del304 no stop* rev where appropriate. The *p3E:2A-tRFP* (provided by Dr. Mary Halloran) contains the coding sequence for the 18-amino acid porcine teschovirus 2A peptide^67^ fused to the start TagRFP (tRFP) (Evrogen). The *pME:Gal4VP16* was from the Tol2Kit^66^. The *pME:tRFP* vector was made by amplifying the tRFP coding sequence from the *p3E:2A-tRFP* vector using *attB1 tRFP* fwd and *attB2r tRFP* rev primers followed by a Gateway BP reaction (Invitrogen). Destination vectors were made with Gateway cloning and the Tol2kit^66^ using *p5E:myl7*, *p5E:CMV/SP6*, *p5E:UAS* as 5′ entry plasmids together with the appropriate pME expression vector and *p3E:2A-tRFP*. These entry vectors were combined with the *pDestTol2pA2* and *pDestTol2CG2* (Tol2Kit), *pDestTol2pACryGFP* or *pDestTol2pACrymCherry* (gifts from Joachim Berger & Peter Currie, Addgene plasmid #64022 and #64023 respectively) as indicated in an LR reaction (Invitrogen).

### Luciferase Assay

HEK293T cells were seeded into a 24-well plate at a density of 1.25 × 10^5^ cells/mL the day before transfection. Transfections were performed using Lipofectamine LTX and PLUS Reagent (Invitrogen). Media was aspirated off and replaced with 500 μL of fresh media containing Lipofectamine LTX, PLUS Reagent, 200 ng of the *pGL2 col2c2-9x luc* plasmid and 50 ng of pBK *CMV:β–galactosidase* plasmid. Additionally, 200 ng of *pDestTol2pA2 CMV:sox9b-2A-tRFP* with or without the *pDestTol2pA2 CMV:dnsox9b-2A-tRFP* were added as indicated and a *pDestTol2pA2 CMV:EGFPpA* plasmid was also included as needed to normalize the mass of added plasmids to 650 ng. Luciferase assays were performed as previously described^68^. A one-way ANOVA was used to compare the means of the groups (n = 4), *p* < 0.05 was considered significant. Results shown are from two independent experiments.

### Zebrafish Husbandry and Transgenic Lines

Adult zebrafish (*Danio rerio*) were housed and maintained according to methods described by Westerfield (2000). The following established transgenic lines were used: *pard3-like*:*EGFP* [*ET*(*krt4*:*EGFP*)^*sqet27*^]^69^, *kdrl*:*GFP* [*Tg(flk:GFP)*^*y1*^]^70^, *fli1a*:*Gal4* [*Tg(fli1a:Gal4FF*^*ubs3*^*;UAS:Kaede)*]^71^. Fertilized eggs were obtained from adult wild-type (AB) or transgenic zebrafish bred in our laboratory as described by Westerfield (2007). Embryos were raised in egg water with 0.003% 1-phenyl-2-thiourea (PTU, Sigma) to inhibit pigment formation. Water changes were made daily. All procedures involving zebrafish were approved by the Institutional Animal Care and Use Committee at the University of Wisconsin-Madison and adhered to the National Institute of Health’s “Guide for the Care and Use of Laboratory Animals”.

### Generation of Transgenic Zebrafish Lines

Newly fertilized eggs were injected with 50 pg of either pDestTol2pACryGFP *myl7:Gal4VP16*, pDestTol2CG2 *UAS:dnsox9b-2A-tRFP*, pDestTol2pACrymCherry *UAS:dnsox9b-2A-tRFP*, pDestTol2CG2 *UAS:tRFP*, or pDestTol2pA2 *myl7:dnsox9b-2A-tRFP* expression plasmids, together with 50–100 pg of Tol2 transposase RNA in Danieau’s solution at the 1–2 cell stage. To generate transgenic lines, injected embryos were screened for expression of the respective transgenesis marker and raised to adulthood where they were then spawned with wild-type AB zebrafish to identify founders with transgene germline incorporation. Zebrafish embryos were raised and screened for germline incorporation of the *Tg(UAS*:*dnsox9b-2A-tRFP)* transgene by crossing to the newly generated *Tg(myl7*:*Gal4VP16)* transgenic zebrafish. Several founders were identified, and the founders giving most complete cardiomyocyte *dnsox9b-2A-tRFP* expression were used for subsequent experiments.

### Microscopy

To visualize the development of heart morphology, the epicardium, and the endocardial cushions, embryos were anesthetized in 0.02% tricaine (MS-222) and mounted in 1.2% low melting point agarose on glass bottom Petri dishes (MatTek). To visualize proepicardium development, embryos were imaged using a camera-mounted stereomicroscope. Confocal imaging was performed on an inverted Nikon A1R scanning laser confocal microscope. Z-stacks spanned approximately 150 μm at 2.825 μm intervals. Live confocal images of the heart were obtained by temporarily exposing mounted embryos to 20 mM BDM (2,3-butanedione 2-monoxime) to inhibit heart contractions long enough to collect each z-stack. Maximum intensity projections were generated using NIS-Elements AR4.30 analysis software and images were processed in Adobe Photoshop and Adobe Illustrator. A Zeiss LSM 880 with Airyscan was used to collect super resolution images of fixed cardiomyocytes. Results show are representative findings from three independent experiments (n = 7–10).

### Immunohistochemistry

Antibody staining was performed as previously described^72^. The antibody against activated leukocyte cell adhesion molecule (Alcam) was used at a 1:25 dilution in PBS with 4% bovine serum albumin and 0.3% Triton (PBT). Rabbit anti-tRFP was obtained from Evrogen and used at 1:200 dilution. Secondary goat anti-mouse antibodies and goat anti-rabbit antibodies (Alexa 633, Alexa 568; Invitrogen) were used at 1:200 dilution in PBT. Embryos were mounted in Vectashield with DAPI (Vector Laboratories).

### Cardiomyocyte cell counts

Ventricular and atrial cardiomyocytes were quantified in fixed *Tg(myl7:Gal4VP16*;*UAS*:*dnsox9b-2A-tRFP)* and (*Tg*(*myl7*:*Gal4VP16*;*UAS*:*tRFP)* samples at 48 and 72 hpf. Samples were mounted in Vectashield with DAPI (Vector Laboratories) to visualize cardiomyocyte cell-bodies. Z-series were collected using a Nikon A1R with a 40x objective lens and a step size of 0.52 microns. Elements software was used to generate maximum intensity projections (MIP) from the z-series. Using Adobe Photoshop, the ventricle and atrium were outlined for each heart in the MIPs and the count tool function used to label and quantify the number of cardiomyocytes in each chamber. Cells were determined to be cardiomyocytes based on the presence of both *myl7*:EGFP and DAPI. Individuals performing the cell counts digitally magnified the image and adjusted levels in the green and blue channels to confirm or rule out colocalization of EGFP and DAPI.

### Cardiac Function

Ventricle volume at end-diastole and end-systole as well as heart rate were measured in *Tg(myl7:Gal4VP16*;*UAS*:*dnsox9b-2A-tRFP)* and *Tg*(*myl7*:*Gal4VP16*;*UAS*:*tRFP)* embryos and larvae. These values were then used to calculate stroke volume, ejection fraction, and cardiac output as described previously^41^. Student’s t-test was used to identify differences between the means of the groups (n = 7), a difference of *p* < 0.05 was considered significant. Results shown are from a single analysis of samples collected on two separate occasions for each timepoint.

### Heart Extraction and Quantitative Real Time Polymerase Chain Reaction (RT-qPCR)

Embryonic hearts were extracted as previously described^44,73,74^. Hearts expressing tRFP were collected from the resulting filtrate using a micropipettor with the aid of an Olympus SZX16 epifluorescence stereomicroscope. Collected hearts were placed into a 10 μL aliquot of culture media and immediately stored in −80 °C until RNA extraction. Heart tissue was gently homogenized in culture media using a plastic pestle then RNA was extracted using Trizol Reagent. 10 µL of RNA, at 11.1 ng/µL, was reverse-transcribed with random hexamers and Multiscribe MuLv from the High Capacity cDNA Reverse Transcription Kit (Thermo Fisher Scientific) per manufacturer’s protocol. 10 µL of the resulting cDNA was preamplified for genes selected for having been characterized as a SOX9 target gene or involved in zebrafish cardiac development, using TaqMan Preamp Mastermix Kit (Thermo Fisher Scientific) for 40 cycles in a 50 µL reaction volume. TaqMan gene expression assays are Minimum Information for Publication of Quantitative Real-Time PCR Experiments (MIQE) compliant and RT-qPCR was performed following MIQE. A list of the TaqMan gene expression assay probes used can be found in Supplemental Table 2. RT-qPCR reactions were performed with the TaqMan Universal Master Mix (Thermo Fisher Scientific) in a 20 µL reaction volume containing 2 µL of the preamplified cDNA. Thermal cycling parameters were carried out per manufacturer’s protocol. Reactions were done in triplicate. RT-qPCR analysis and calculations were performed in the Sequence Detection System v 2.4. Several genes were assessed to determine the best reference gene to be used for this data set (data not shown). *actb1* was found to be the most consistent, not altered between *Tg(myl7*:*Gal4VP16;UAS:dnsox9b-2A-tRFP)* and *Tg(myl7:Gal4VP16;UAS:tRFP)*, and showed the least variance in this analysis. All transcripts examined (Supplemental Table 2) were normalized to *actb1* through the comparative C_t_ (ΔΔ Ct) method. A one-tailed Student’s t-test with a Benjamini-Hochberg multiple-comparison correction^75^ (FDR < 0.25) was used to identify differences between the means of the two groups (n = 4), a p-value < 0.05 was considered significant. Results shown are from a single analysis of samples collected on at least four different occasions.

### Prediction of Transcription Factor Binding Sites

DNA sequences from the zebrafish genome (GRCz11/danRer11) were obtained using the University of California, Santa Cruz (UCSC) Genome Bioinformatics site (http://genome.ucsc.edu). We examined whether SOXE protein binding sites (SOX8, SOX9, and SOX10) were present in the 2.5 kb upstream region relative to the transcriptional start site for the following genes: *capns1a* (NM_ 001017899), *c-fos* (NM_ 205569), *myl7* (NM_ 131329), *nkx2.5* (NM_ 131421), and *nkx2.7* (NM_ 131419) genes. SOXE protein binding sites were predicted with the MatInspector program (Genomatix Software GmbH, Munich, Germany) using a Matrix Similarity Score >0.75.

## Electronic supplementary material

Supplemental Materials

Supplemental Movie 1. Control 48 hpf

Supplemental Movie 2. Control 72 hpf

Supplemental Movie 3. dnsox9b 48 hpf

Supplemental Movie 4. dnsox9b 72 hpf

Supplemental Movie 5. dnsox9b 72 hpf

Supplemental Movie 6. dnsox9b 72 hpf

## Data Availability

The datasets generated during and/or analyzed during the current study that are not published in this article are available from the corresponding author on reasonable request.
