# Supplementary material for: sox9b is required in cardiomyocytes for cardiac morphogenesis and function
Source: Sci Rep. 2018 Sep 17;8:13906. doi: 10.1038/s41598-018-32125-7 (PMC6141582; doi:10.1038/s41598-018-32125-7)
Supplement: Supplementary file 1 — Supplemental Materials [file 41598_2018_32125_MOESM1_ESM.docx]

***sox9b* is required in cardiomyocytes for cardiac morphogenesis and function**

Joseph C. Gawdzik^1,2^, Monica S. Yue^1,2^, Nathan R. Martin^5^, Loes M. H. Elemans^2,3^, Kevin A. Lanham^2^, Warren Heideman^1,2^, Ryan Rezendes^5^, Tracie R. Baker^4^, Michael R. Taylor^2^ and Jessica S. Plavicki*^5^

^1^Molecular and Environmental Toxicology Center, University of Wisconsin at Madison, Madison, WI, USA

^2^Division of Pharmaceutical Sciences, University of Wisconsin at Madison, Madison, WI, USA

^3^Division of Toxicology, Institute for Risk Assessment Sciences (IRAS), Utrecht University, Utrecht, NL

^4^Wayne State University, Institute of Environmental Health Sciences, Detroit, MI, USA

^5^ Department of Pathology and Laboratory Medicine, Brown University, Providence, RI, USA

*To whom correspondence should be addressed: [jessica_plavicki@brown.edu](mailto:jessica_plavicki@brown.edu)

Tel: (401) 863-6112

Brown University, Department of Pathology and Laboratory Medicine, 70 Ship Street, Providence, RI, 02703

**Running title:** Essential functions for *sox9b* in cardiomyocytes

Supplemental Figure 1. Inhibition of *sox9b* function in cardiomyocytes does not alter the development of proepicardial progenitor cells.

Proepicardium development was examined in control (*Tg(myl7:Gal4VP16;UAS:tRFP)*) larvae and larvae with cardiomyocyte-specific loss of *sox9b* expression (*Tg(myl7:Gal4VP16;UAS:dnsox9b-2A-tRFP))*. 72 hpf larvae were mounted in methylcellulose and imaged using camera mounted on a stereomicroscope. The proepicardium formed in all zebrafish larvae examined (n=7) and can be seen in the above image, outlined in white. Ventricle (V), Atrium (At) and Proepicardium (PE) are abbreviated as indicated. Anterior to the left.

Supplemental Movie 1. A control zebrafish heart at 48 hpf. Brightfield movie of a control embryo (*Tg(myl7:Gal4VP16;UAS:tRFP)*) at 48 hpf. 20x magnification.

Supplemental Movie 2. A control zebrafish heart at 72 hpf. Brightfield movie of a control larva (*Tg(myl7:Gal4VP16;UAS:tRFP)*) at 72 hpf. 20x magnification.

Supplemental Movie 3. Cardiomyocyte-specific inhibition of sox9b function disrupts cardiac function at 48 hpf. Brightfield movie of an embryo with cardiomyocyte-specific inhibition of *sox9b* function (*Tg(myl7:Gal4VP16;UAS:dnsox9b-2A-tRFP))* at 48 hpf. 20x magnification.

Supplemental Movies 4-6. Cardiomyocyte-specific inhibition of sox9b function disrupts cardiac function at 72 hpf. Brightfield movie of an larva with cardiomyocyte-specific inhibition of *sox9b* function (*Tg(myl7:Gal4VP16;UAS:dnsox9b-2A-tRFP)*) at 72 hpf. A hole is clearly present in the atrium in Supplemental Movie #4. Holes were observed in both the atrium and ventricle, but were not present in all samples. Clustering and pinching of endocardial cells in the atrium in visible in Supplemental Movies #4 and 5. 20x magnification.


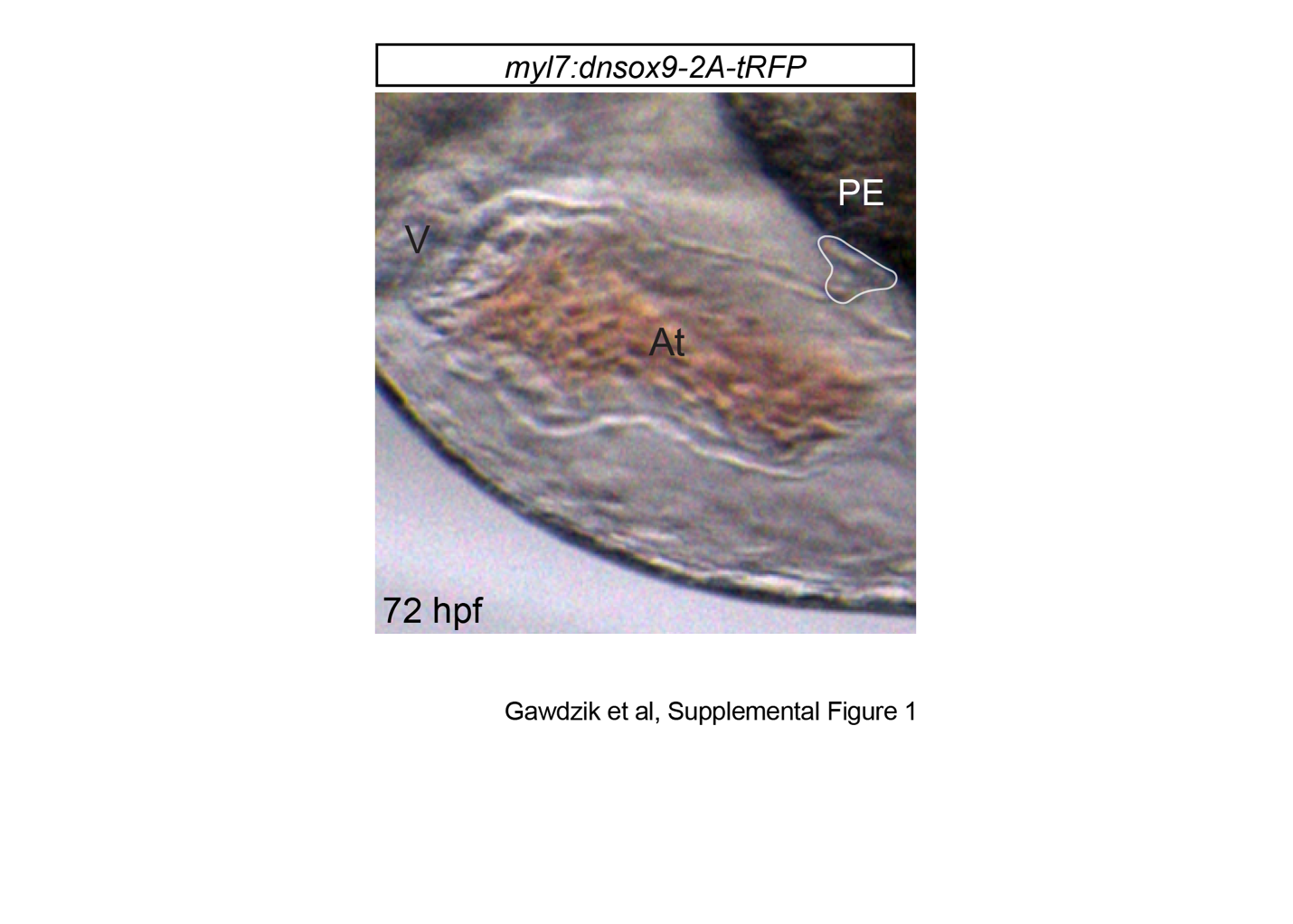


Supplemental Table 1. Primer sequences

| **Name** | **Sequence** |
| --- | --- |
| pGL2 fwd | 5’--TGT ATC TTA TGG TAC TGT AAC TG--3’ |
| pGL2 col2c2-3x fwd | 5’--GCT GCT CGA GAT CTA AAG CC--3’ |
| pGL2 col2c2-3x rev | 5’--CCA AGC TTA CTT GGA TCC ATC TC--3’ |
| E1b minimal promoter  HindIII fwd | 5’--CGC AGT AAG CTT GGT CGA CTC TAG AGG G--3’ |
| E1b minimal promoter  HindIII rev | 5’--CGC TCC GCA AGC TTG AAC TAG TGG AT--3’ |
| attB1 sox9b ORF fwd | 5’--GGG GAC AAG TTT GTA CAA AAA AGC AGG CTG CCA CCA TGA ATC TCC TCC AGC GC--3’ |
| attB2 sox9b no stop rev | 5’--GGG GAC CAC TTT GTA CAA GAA AGC TGG GTG GGG TCT GGA CAG CTG TGT GTA GAC G--3’ |
| attB2 sox9b del304  no stop rev | 5’--GGG GAC CAC TTT GTA CAA GAA AGC TGG GTC GGC TGC GCT GCC GTA CCC--3’ |
| attB1 tRFP fwd | 5’--GGG GAC AAG TTT GTA CAA AAA AGC AGG CTG CCA CCA TGG TGT CTA AGG GCG AAG--3’ |
| attB2 tRFP rev | 5’--GGG GAC CAC TTT GTA CAA GAA AGC TGG GTC TCA ATT AAG TTT GTG CCC CAG--3’ |

Supplemental Table 2. Genes analyzed in RT-qPCR assay

| **Symbol** | **Gene Name** | **NCBI  Reference  Sequence** | **TaqMan® Gene  Expression  Probe ID** | **Experimental  Rationale** | **Reference** | **Tissues  Examined** |
| --- | --- | --- | --- | --- | --- | --- |
| *atf3* | *activating  transcription  factor 3* | NM_200964.1 | Dr03100567_m1 | SOX9 Target | Ohba  et al. (2015) | Mouse  Chondrocytes |
| *capns1a* | *calpain, small  subunit 1 a* | NM_001017899 | Dr03424055_m1 | SOX9 Target | Ohba  et al. (2016) | Mouse  Chondrocytes |
| *col1a2* | *collagen, type I,  alpha 2* | NM_182968.2 | Dr03074437_m1 | SOX9 Target | Garside  et al. (2015) | Embryonic  Mouse Limb |
| *col2a1a* | *collagen, type II,  alpha 1a* | NM_131292.1 | Dr03099263_m1 | SOX9 Target | Bell  et al. (1997) | Mouse  Chondrocytes |
| *fn1b* | *fibronectin 1b* | NM_001013261.1 | Dr03141116_m1 | SOX9 Target | Ohba  et al. (2016) | Mouse  Chondrocytes |
| *fos* | *c-Fos* | NM_205569.1 | Dr03100810_g1 | SOX9 Target | Ohba  et al. (2017) | Mouse  Chondrocytes |
| *hapln1a* | *hyaluronan,  proteoglycan  link  protein 1a* | NM_001007790.2 | Dr03147484_m1 | SOX9 Target | Ohba  et al. (2018) | Mouse  Chondrocytes |
| *hdac1* | *histone  deacetylase 1* | NM_173236.1 | Dr03099534_m1 | SOX9 Target | Garside  et al. (2015) | Embryonic  Mouse  Heart Valve |
| *matn4* | *matrilin 4* | NM_213549.1 | Dr03114677_m1 | SOX9 Target | Ohba  et al. (2015) | Mouse  Chondrocytes |
| *twist1b* | *twist family  bHLH  transcription  factor 1b* | NM_001017820.1 | Dr03423977_g1 | SOX9 Target | Garside  et al. (2015) | Embryonic  Mouse  Heart Valve |
| *myh6* | *myosin,  heavy chain 6,  cardiac muscle,  alpha* | NM_198823.1 | Dr03112899_s1 | Cardiac  Development | Shih  et al. (2015) | Myocardium |
| *myl7* | *myosin,  light chain 7,  regulatory* | NM_131329.3 | Dr03105700_m1 | Cardiac  Development | Yelon  et al. (1999) | Myocardium |
| *nkx2.5* | *NK2 homeobox 5* | NM_131421.1 | Dr03074126_m1 | Cardiac  Development | Tu  et al. (2009) | Myocardium |
| *nkx2.7* | *NK2 transcription  factor related 7* | NM_131419.1 | Dr03150332_m1 | Cardiac  Development | Tu  et al. (2010) | Myocardium |
| *vmhc* | *ventricular myosin  heavy chain* | NM_001112733.1 | Dr03431136_m1 | Cardiac  Development | Yelon  et al. (1999) | Myocardium |
| *sox9b* | *SRY-box 9b* | NM_131644.1 | Dr03080049_m1 | Cardiac  Development | Hofsteen  et al. (2013) | Myocardium |
| *sox9a* | *SRY-box 9a* | NM_131643.1 | Dr03112283_m1 | Homolog | Chiang  et al. (2001) | Myocardium |
| *actb1* | *actin, beta 1* | NM_131031.1 | Dr03432610_m1 | Reference  Gene | --- | --- |

Supplemental Table 3. dnSox9b target gene relative expression

| **Target  Gene** | **Average relative expression normalized to *actb1*** | | **p-value Student's t-test one-tailed** | **Multiple Comparison Test Benjamini-Hochberg FDR < 0.25** | **Significant** |
| --- | --- | --- | --- | --- | --- |
|  | **Control** | **dnsox9b** |  |  |  |
| *myl7* | 8.1271 | 3.9596 | 0.0004 | 0.0075 | Yes |
| *nkx2.7* | 0.1293 | 0.0668 | 0.0144 | 0.1226 | Yes |
| *fos* | 1.8540 | 0.6402 | 0.0144 | 0.0818 | Yes |
| *capns1a* | 0.0726 | 0.0300 | 0.0154 | 0.0655 | Yes |
| *nkx2.5* | 0.0618 | 0.0426 | 0.0219 | 0.0745 | Yes |
| *col2a1* | 0.0118 | 0.0248 | 0.1157 | 0.3279 | No |
| *col1a2* | 0.2005 | 0.1036 | 0.1722 | 0.4183 | No |
| *twist1b* | 0.0296 | 0.0224 | 0.1842 | 0.3914 | No |
| *hdac1* | 0.1815 | 0.2044 | 0.2679 | 0.5061 | No |
| *halpn1a* | 0.0764 | 0.0672 | 0.3094 | 0.5260 | No |
| *matn4* | 0.0931 | 0.0804 | 0.3211 | 0.4963 | No |
| *sox9a* | 0.0521 | 0.0602 | 0.3383 | 0.4792 | No |
| *atf3* | 0.0776 | 0.0823 | 0.3861 | 0.5050 | No |
| *myh6* | 1.3466 | 1.2347 | 0.3993 | 0.4848 | No |
| *fn1b* | 0.0064 | 0.0072 | 0.4035 | 0.4573 | No |
| *vmhc* | 6.2515 | 6.4691 | 0.4594 | 0.4594 | No |
